# Supplementary material for: Splice-Junction-Based Mapping of Alternative Isoforms in the Human Proteome
Source: Cell Rep. Author manuscript; Available in PMC 2020 Jan 15. (PMC6961840; doi:10.1016/j.celrep.2019.11.026)
Supplement: 2 [file NIHMS1546469-supplement-2.zip › DF1/PXD010154/Testis/Testis_37_ZNF512_EPPVYAAGNVYLSSLWETTSFTGR.pdf]

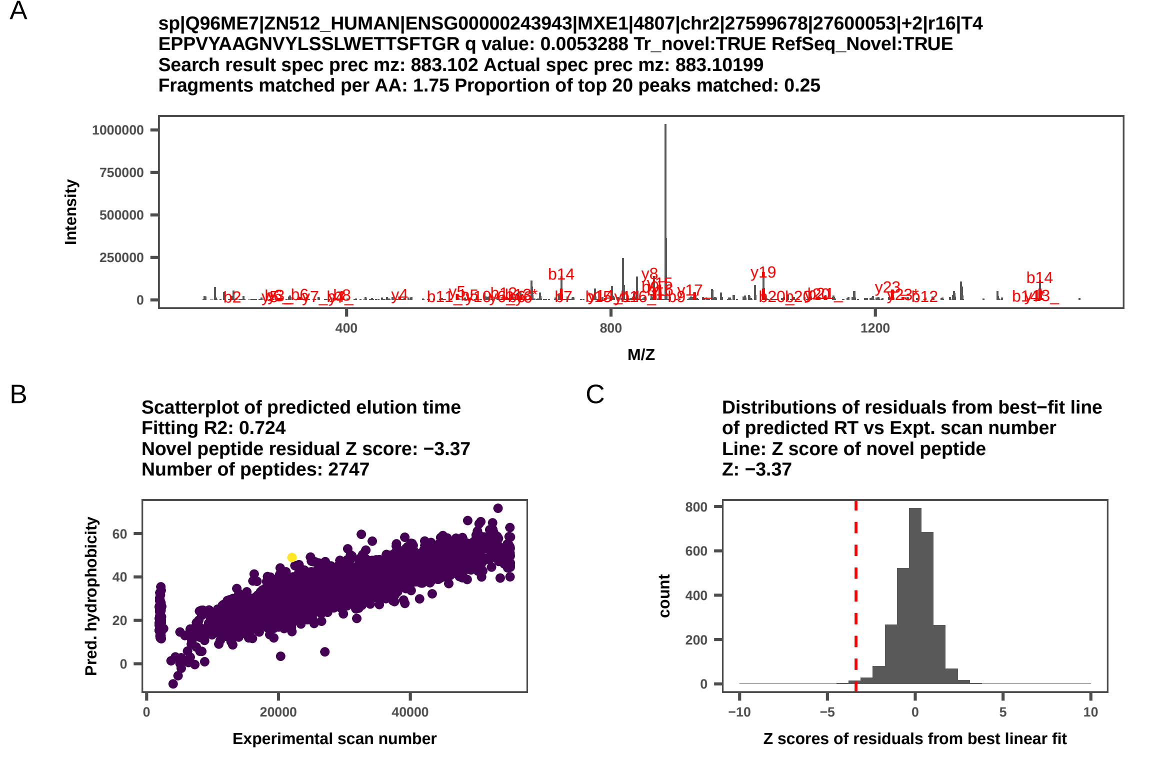

A
sp|Q96ME7|ZN512_HUMAN|ENSG00000243943|MXE1|4807|chr2|27599678|27600053|+2|r16|T4
EPPVYAAGNVYLSSLWETTSFTGR q value: 0.0053288 Tr_novel:TRUE RefSeq_Novel:TRUE
Search result spec prec mz: 883.102 Actual spec prec mz: 883.10199
Fragments matched per AA: 1.75 Proportion of top 20 peaks matched: 0.25
Intensity
1000000
750000
500000
250000
0
b14
y8
y19
b14
b2
y4
b7
b9
y17
y23
400
800
1200
M/Z
B
Scatterplot of predicted elution time
Fitting R2: 0.724
Novel peptide residual Z score: −3.37
Number of peptides: 2747
Pred. hydrophobicity
60
40
20
0
0
20000
40000
Experimental scan number
C
Distributions of residuals from best−fit line
of predicted RT vs Expt. scan number
Line: Z score of novel peptide
Z: −3.37
count
800
600
400
200
0
−10
−5
0
5
10
Z scores of residuals from best linear fit
